# A Deep Neural Network Sensor for Visual Servoing in 3D Spaces

**DOI:** 10.3390/s20051437

**Published:** 2020-03-06

**Authors:** Petar Durdevic, Daniel Ortiz-Arroyo

**Affiliations:** Department of Energy Technology, Aalborg University, Niels Bohrs Vej 8, 6700 Esbjerg, Denmark

**Keywords:** deep convolutional neural network, visual servoing, drone, inspections, autonomy

## Abstract

This paper describes a novel stereo vision sensor based on deep neural networks, that can be used to produce a feedback signal for visual servoing in unmanned aerial vehicles such as drones. Two deep convolutional neural networks attached to the stereo camera in the drone are trained to detect wind turbines in images and stereo triangulation is used to calculate the distance from a wind turbine to the drone. Our experimental results show that the sensor produces data accurate enough to be used for servoing, even in the presence of noise generated when the drone is not being completely stable. Our results also show that appropriate filtering of the signals is needed and that to produce correct results, it is very important to keep the wind turbine within the field of vision of both cameras, so that both deep neural networks could detect it.

## 1. Introduction

Our physical surrounding environment has a huge amount of information that is constantly perceived by our vision system. Our brain has learned to localize ourselves while we move within an unknown physical space, recognizing the objects contained in it effortlessly. However, providing the same capabilities in unmanned autonomous vehicles (UAV) is challenging. Recent advances in machine learning and computer vision for object recognition, object detection and probabilistic localization have made possible to design UAVs capable of emulating how humans perceive the environment, achieving different degrees of autonomy.

In object recognition, an image is analyzed to identify if it contains certain classes of objects. In object detection, objects are not only identified but its location within an image is also determined. Classical approaches for object recognition and detection in computer vision have employed a variety of methods, from sliding windows [1], boosted object detectors [2] to Histograms of Oriented Gradient (HOG) descriptors [3]. However, more recent approaches use deep learning techniques [4,5,6,7,8,9] optimized to achieve higher accuracy while working in real-time.

Among unmanned vehicles, drones are very popular due to their low cost and flexibility of use. Drones are commonly applied in many domains such as surveillance and monitoring, inspection, goods delivery etc. One of the drone applications that are the main focus of this paper, is wind turbine inspections. In this application, drones are made to fly at a safe distance from the wind turbine’s blades to take images and search for possible damages. In today’s inspections, drones are operated manually by an expert pilot who constantly has to monitor the camera and the drone movements looking for damages. To replace the manual inspections, an autonomous drone, first, needs to have the capability of locating a wind turbine in the surrounding area and navigate towards its hub, following a straight path. The hub is usually located on top of the tower and is the pivoting point for the three blades. Once the drone is close, it should follow a predefined path with respect to each of the blades, maintaining a safe distance from them.

Global positioning system (GPS) data can be used for UAV’s localization and navigation in outdoor environments. However, in wind turbine inspections a drone cannot rely on using only GPS data, due to its inaccuracy, in particular regarding the altitude. Moreover, the localization relates to the tower’s position, i.e., the north and east coordinates, but not to the exact three-dimensional location of the hub which can be located 360 degrees around the tower’s axis.

Real-time kinematic (RTK) positioning, substantially improves localization accuracy over that provided by GPS, but it is still expensive and requires a special set up of the equipment that needs to be installed in each turbine. Additionally, RTK requires special calibration and the implementation of a communication protocol between a ground station and the drone. Lastly, a number of Simultaneous Localization and Mapping (SLAM) algorithms have been proposed in the literature [10] that construct, using different types of sensors, a two dimensional (2D) or three dimensional (3D) map as the UAV is moving. This map allows the UAV to localize itself and navigate within an unknown environment. SLAM [11] is capable of mapping the environment and identifying landmarks, but is not able to infer, from this data, what kind of object it is observing.

Another option to implement a drone’s navigation system is to use visual servoing. In visual servoing, data from the camera sensor is fed back to control the position of a robot [12]. Visual servoing in 3D allows a drone to move within an open space towards a target object by using its video camera. Several implementations of visual servoing have been proposed in the literature. Some approaches employ road or trail track recognition to guide the drone [13], other approaches first learn the environment [14,15]. However, in on-shore or off-shore wind turbine installations, navigation is more challenging since there are no tracks or landmarks that could be used as guidance for a drone.

Deep neural networks (DNNs) have made possible to recognize classes of objects from the rich visual information of a camera. In this work, we leveraged the advantages of using DNNs for object detection and localization to create a novel vision sensor that is capable of determining the position of an object in the real physical space using only a stereo camera. Our sensor employs DNNs to localize the 2D position of a wind turbine in the image plane represented as a vector and then compute the 3D coordinates of the wind turbine. We have performed a group of experiments to characterize our visual sensor and determine its applicability to a visual servoing system. The ultimate goal is that our sensor together with a controller specially designed for the purpose, could be used as the main navigation system for a drone designed to perform wind turbine inspections autonomously.

The sensor described in this paper is the whole vision system consisting of a stereo camera and a DNN attached to each camera.

The contributions made by this paper are twofold. First, to the best of our knowledge, there is no other similar vision-based sensor that employs the techniques proposed in this paper. Secondly, no other paper has attempted to characterize the performance of this type of vision-based object detection system used as a sensor. This paper is our first step in this direction.

The paper is structured as follows: Section 2 provides a brief review of the state of the art, Section 3 describes current related work, Section 4 introduces the DNN’s design used in our sensor and its training, Section 5 introduces the epipolar image analysis and projective geometry and the fusion with the DNNs for correspondence estimation, Section 6 describes the experimental setup, Section 7 presents the results of the experiments, Section 8 discusses our solution, and Section 9 concludes the paper.

## 2. State of the Art in Visual Servoing

Visual servoing for UAVs was implemented in [13] employing a deep neural network (DNN) for visual perception of forest trails. A monocular image is an input to the DNN and the output consists of three values which are the probabilities of three classes of movements [Turn left, Go straight, Turn right]. The training dataset was collected via three head-mounted cameras. Similarly, in [14] a drone for autonomous navigation of indoor corridors is presented. The drone’s camera captures images that are sent to a control station for processing. The control station employs a DNN called DenseNet-16, that was previously trained to classify corridor images as center, left or right. The DNN controls drone navigation by learning safe navigation strategies from the extracted images and sending commands to the drone.

Another work reported in [16] targets trail following. A micro aerial vehicle (MAV) uses a DNN called TrailNet to determine the orientation [facing left, facing center, facing right] and lateral offset [shifted left, centered, shifted right] of the MAV with respect to the center of the trail. This data is used to compute MAV’s pose and control its movements. An additional DNN that is coupled with a visual odometry component, is used for obstacle detection and avoidance.

Another example of a drone that employs convolutional neural networks (CNN) is DroNet, a drone reported in [15] that travels safely through the streets of a city. DroNet contains an eight-layers residual network that produces for each input image, a steering angle that keeps the drone navigating while it avoids obstacles, and a collision probability to let the UAV recognize and avoid dangerous situations. DroNet is trained with data collected by cameras mounted on cars and bicycles.

DNNs have also been used to allow ground vehicles navigate autonomously in roads. A recent approach described in [17], uses a pre-trained CNN to map pixels directly from a single front camera mounted in the vehicle to steering wheel commands. The system learns to drive in traffic with or without lane markings in roads and highways. This system is called end-to-end because instead of using lane detection, path planning and control algorithms separately, it optimizes all this processing simultaneously using the representation model learned by CNN. Refs [13,15,16,18,19,20,21,22,23] could be classified as end-to-end methods.

Similar, but more recent works in [22,24], presented frameworks comprising of a combination of techniques to make a ground AUV drive at high speeds using a monocular camera together with an inertial measurement unit (IMU) and wheel speed sensors. This system used a combination of DNNs, particle filters, and model predictive control (MPC). Deep CNNs, combined with long short term memory (LSTM) networks, were used to learn a local cost map representation of the road. The particle filters computed the AUV’s localization and the MPC controlled the vehicle while driving at high speeds.

The work described in [25] uses a ground robot and MobileNet [26] implementation of Single Shot Multibox Detector (SSD) [9] architecture to keep track of a person, making sure that the target remains in the field of vision of the camera, as it is moving. This is done by calculating a proportional-integral (PI) control law that drives a ground robot equipped with a camera that keeps track of the moving target. A set-point bounding box position placed at the center of the image is compared to the position of the bounding box produced by MobileNet when a person is detected. The robot moves to keep camera’s forward speed and yaw rate at a specified safe distance from the moving target object.

In [27] a 3D object detection system using stereo vision for autonomous driving is described. The images from two cameras are the inputs to two Region-based Convolutional Neural Networks (RCNN) [4]. Extra layers were added to the R-CNNs to detect sparse keypoints, viewpoints and dimensions. The network achieved 30% improvement over other stereo-based systems on the KITTI dataset.

In [28] a visual servoing system for the pose estimation of a gantry robot is presented. The system uses transfer learning to retrain a CNN called Alexnet to perform relative camera pose estimation for the robot with 6 degrees of freedom. Alexnet [29] is a deep CNN that ignited the interest of the machine learning community when it achieved breakthrough performance on the ILSVRC 2010 image recognition contest. In [28] the last classification layer of Alexnet was substituted by a regression layer using transfer learning techniques [30]. This produced as output the six degrees of freedom of a pose. The network was trained using a synthetic dataset containing multiple views, illumination changes and added occlusions. The system achieved millimeter accuracy in pose estimation.

Our previous works on visual servoing for drones also used Alexnet. For instance, in [23], we used transfer learning changing the last layers of Alexnet to create a network capable of recognizing wind turbines. The DNN was called WindMillNet and its output was the probability that an image from the camera contained a wind turbine. This probability was used to change the state of the controller in a drone switching from its scanning state, while it searched for a wind turbine to a found state where the drone navigated autonomously forward, towards the wind turbine.

Later in [31] we created WindTurbineNet, a DNN that had the same structure as WindMillNet, but the final layer was changed, to allow WindTurbineNet recognizing the four classes of objects that were part of the lab’s experimental setup i.e., [curtain; net; wall; windturbine]. In that work we split the images coming from drone’s camera into four equally sized segments, to make the drone navigate autonomously toward a wind turbine. Then, we attached to each segment a DNN that calculated the probability that it contained a wind turbine. The upper two probabilities were then fused and used in the visual servoing control system to make the drone navigate left or right according to the probability value obtained.

## 3. Current Work

This paper is a continuation of our previous work in [23], where we used image segmentation for visual servoing. In this work, we avoid segmentation of the video signal and instead use YOLO9000 or Yolov2 as an augmented sensor for localization of objects in the environment. Yolov2 allows us to determine the location and size of objects in the perspective projection of the environment.

To do this, two Yolov2 networks were attached to the stereo camera to localize the wind turbine’s position in the 3D world coordinate system, using epipolar-plane image analysis. Using the DNNs we solved the correspondence problem in epipolar-plane image analysis.

The works reported in [25,27], are closest to the one presented in this paper. They both use DNNs for object detection, but there are several important differences.

First, we use a camera with stereo vision instead of a monocular camera as is done in [25]. Contrary to [25], our system uses Yolov2 [7] trained to localize wind turbines with bounding boxes from camera images, instead of tracking moving objects.

Secondly, our system is not designed to detect 3D objects as was done in [27] but we use 2D stereo vision object detection data to determine the distance from drone’s camera to the real physical object. This was done by attaching a Yolov2 DNN to each camera and computing the distance by stereo triangulation techniques, using a similar technique to the one in [32].

## 4. Deep Neural Network

Yolo comprises a family of DNNs designed for detecting objects from camera images in real-time. The original version of Yolo, described in [6], is capable of detecting objects of up to 1000 different classes, enclosed within bounding boxes in images. Yolo includes a CNN with 22 layers that were pre-trained on the 1000 classes on ImageNet dataset. Yolo divides the input image normally into a 7×7 grid of cells. Each grid cell commonly predicts two bounding boxes jointly with the confidence scores indicating that a box contains an object of a class. The confidence score is a function of the ratio between the intersection over the union (IOU) function between the bounding box and the ground truth areas. To speed up computation, boxes with low probability values or that are redundant because they have the highest shared area with others, are removed. For object detection, Yolo adds four convolutional and two fully connected layers with weights randomly initialized. The original Yolo DNN was capable of operating in real-time but suffered from low detection precision and was relatively slow, reaching a detection speed of 45 frames per second (FPS).

The next version of Yolo, called Yolo9000 or Yolov2 [7], was designed to improve object detection precision and speed over the original Yolo, surpassing even other object detection networks such as SSD [9] and R-CNN [4]. The last version of Yolo, called Yolov3 in [8] was an incremental improvement over Yolov2 aimed at improving the real-time execution speed.

In this paper, we used Yolov2 as our object detection network. However, any other object detection DNN will also work. The main reason for choosing Yolov2 was its availability and the fact that to characterize the sensor, we did not need the faster inference speed provided by Yolov3. Yolov2’s architecture is similar to the original Yolo but includes several important improvements. Some of them are batch normalization, a higher resolution classifier with size 448×448, training with images of multiple scales, clustering algorithms to predict the box center and dimensions and the use of fine-grained features. Yolov2 is capable of recognizing 9000 classes of objects, reaching 78.6 of mean average precision (mAP) at 40 FPS on the VOC 2007 data-set when trained with image sizes of 544×544 on the combined COCO and ImageNet datasets.

Yolov2 is composed of two main sub-networks. The first sub-network is a feature extracting network. This sub-network could be any convolutional neural network (CNN) that has been pre-trained on a data-set of images. Examples of CNNs that can be used for this purpose are AlexNet, GoogleNet or RestNet50. The detection sub-network comprises all the layers that Yolov2 requires to detect objects within bounding boxes and the layers used to improve precision and accuracy with respect to the original version of Yolo.

In the rest of the paper, we will use the name YOLO to identify the Yolov2 DNN that is used in this work.

### 4.1. Data Acquisition, Augmentation and Training

Our version of YOLO uses RestNet50 as the feature extraction network. RestNet50 is a CNN with 50 layers that were pre-trained on the ImageNet data-set. Layer 40 in RestNet50 was chosen as the start of the detection sublayer. This layer uses rectified linear units (RELU) as activation functions and the feature maps produced by the preceding feature extraction sub-network were downsampled by a factor of 16 to reduce the map’s dimensionality. The rest of the layers comprise Yolov2’s object detection and optimization layers. Our network has a total of 150 layers.

To train YOLO we used a low learning rate of 0.001, so that RestNet50 does not forget the basic features already learned from the ImageNet dataset.

**Figure 1:**
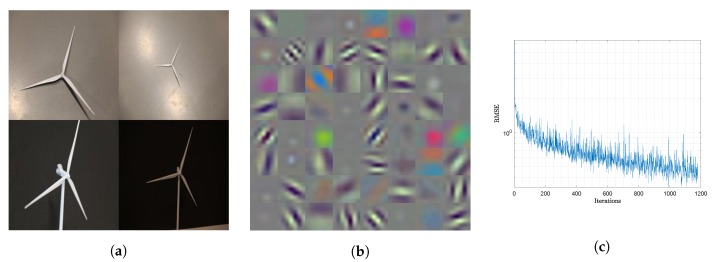
Partial output of feature extraction sub-network, examples of training images and training results. (**a**) Training images of wind turbines; (**b**) Output feature maps of 2nd layer; (**c**) RMSE when training for 10 epochs and 1180 iterations.

The training data-set we used comprised 590 images of wind turbines with sizes 224×224×3, the minimum image size supported by YOLO. An example of four images is shown in Figure 1a. Each image was manually labeled with the ground truth bounding boxes placed around the wind turbine blades. Using small images helps the network to capture the main global features of an object but at the cost of missing finer details. Larger image sizes help to capture the finer features but at the cost of missing the global ones and increasing training time. Since in this paper we are interested in capturing the global features of a wind turbine a small image size was used.

Finally, we increased the training dataset using synthetic data augmentation by applying horizontal flipping, random scaling and color jittering to create a final training dataset of 1182 images. These simple image transformation methods were used so that the original ground truth bounding boxes that were inserted manually, could be easily recomputed for the new transformed images, at run time.

Figure 1b shows the output feature maps in the second layer of the feature extraction sub-network in RestNet50. The figure shows that after training, the feature extraction sub-network was capable of extracting the basic patterns of wind turbine’s blades from the training image data-set.

We trained the network with stochastic gradient descent in 10 epochs and 1180 iterations on a NVidia GTX1060 graphics processing unit (GPU). Figure 1c shows how the root mean square error (RMSE) decreases on average each epoch, reaching a minimum of 0.42.

YOLO requires an initial set of anchor boxes that are used by the network as priors, to adjust their sizes when a target object is detected in part of an image. To compute the initial size of these priors the k-means algorithm was applied on the whole set of bounding boxes that were manually drawn on the images of the training data-set. K was set to 4, to computing four clusters that were used to calculate the dimensions of the anchor boxes. The training bounding boxes were clustered using the intersection over union (IOU) measure as a distance metric.

### 4.2. YOLO Output

**Figure 2:**
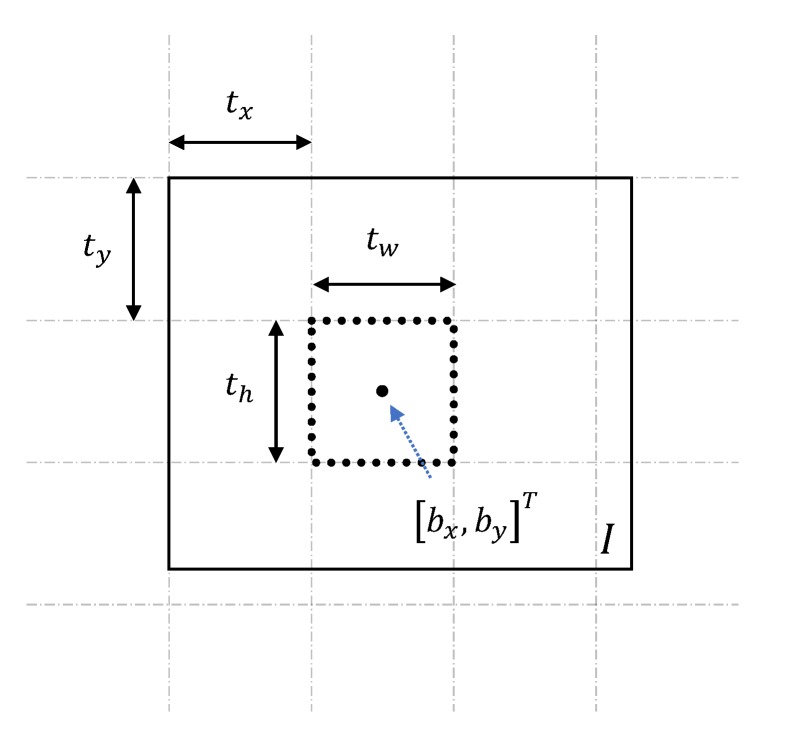
Sketch of the bounding box.

YOLO computes bounding boxes and outputs the following four parameters:

(1) $${\left[\begin{array}{c} t_{x},t_{y},t_{w},t_{h} \end{array}\right]}^{T},$$

which is the location of the *x* and *y* offset and the width and height of the bounding box, respectively, as illustrated in Figure 2.

To navigate toward the center of the wind turbine, we compute the center of the bounding box by performing the following calculations:

(2) $$\begin{array}{c} b_{x}=t_{x}+\frac{t_{w}}{2}\\ b_{y}=t_{y}+\frac{t_{h}}{2}. \end{array}$$

## 5. Augmented Visual Servoing Sensor

The following section describes the methods applied to compute the 3D position of the scene point *P*. Before the method is described several assumptions about the camera were made to simplify the modeling.

**Assumption** **1.** *The lens are parallel to the sensor and thus there are no misalignment of the u and the v axis.*

**Assumption** **2.** *The camera lens has no radial distortion*

**Assumption** **3.** *A thin lens camera is used, leading to the applicability of the pinhole camera model*

**Assumption** **4.** *Both cameras have the same calibration*

**Assumption** **5.** *The scene point is visible to both cameras*

### 5.1. Perspective Projection

**Figure 3:**
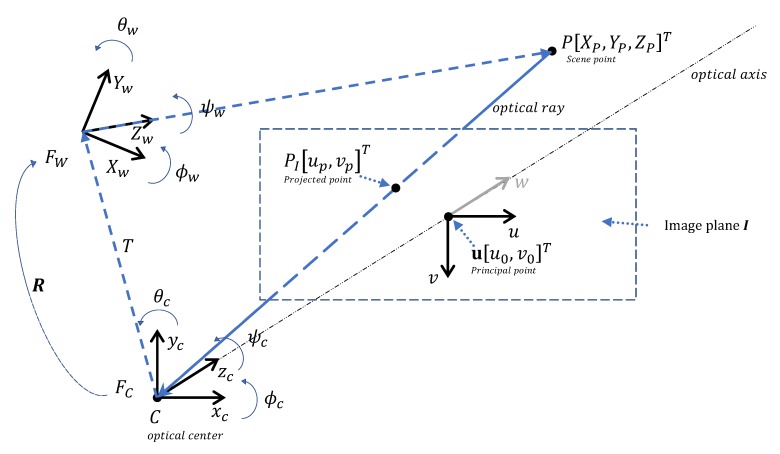
Perspective projection.

When the drone is moving in a three dimensional (3D) space, an observed point has coordinates $\left[X_{w},Y_{w},Z_{w}\right]$ in the world reference frame $F_{W}$ (We use capital letters for the world frame coordinates.). In our case the observation occurs through the camera sensor located in the reference frame $F_{C}$ having the origin *C* with coordinates ${\left[x_{c},y_{c},z_{c}\right]}^{T}$, which is the center of projection, where the *Z* axis is the optical axis. The image sensor is intrinsically two-dimensional (2D), and is located in the image reference frame $F_{I}$. The projection of the scene in the sensor contains the ‘real’ information in the form of light waves, which are converted into a 2D matrix of values. Each matrix contains information on the lightness and color of one small area of the scene. This is called the intensity image [33]. Each element of the image matrix is referred to as a pixel with coordinates ${\left[u_{pixel},v_{pixel}\right]}^{T}$. Each pixel has width and height, that, depending on the focal length and the distance to the object, is proportional within some scale to the ‘real’ world image. Thus, any observation of the ‘real’ world through the camera lens will be subjected to a conversion from 3D to 2D space through a perspective projection, as illustrated in Figure 3.

When the drone observes a scene point *P* in $F_{W}$ with coordinates ${\left[x_{P},y_{P},z_{P}\right]}^{T}$, it is projected onto the image plane *I* and its projection is $P_{I}$ with the following coordinates ${\left[u_{p},v_{p}\right]}^{T}$. Due to assumptions 1 and 2, $F_{I}$ coincides with the camera reference frame $F_{c}$. If we assume that the world frame and the camera frame are aligned, the coordinates of $P_{I}$ can be derived from similar triangles relation [33]:

(3) $$\begin{array}{c} u_{p}=\frac{f}{Z}X_{P},\\ v_{p}=\frac{f}{Z}Y_{P}, \end{array}$$

where *f* is the focal length, *Z* is the distance from the principal point **u** to *P*, according to Figure 3.

### 5.2. Camera Calibration

Camera calibration involves the estimation of the extrinsic parameters and intrinsic parameters.

#### 5.2.1. Extrinsic Parameters

The fundamental equations for the perspective projection (3) are represented in the camera reference frame, which is not known. We aim at knowing its location relative to the world reference frame. The extrinsic parameters allow the transformation from the known world reference frame, to the camera reference frame which is unknown [34]. The transformation is illustrated in Figure 3.

After this, the relationship between world’s reference frame to the camera’s reference frame is expressed in Equation (4), [34].

(4) $$\left[\begin{array}{c} x_{c}\\ y_{c}\\ z_{c} \end{array}\right]=R\left[\begin{array}{c} X_{w}\\ Y_{w}\\ Z_{w} \end{array}\right]+T,$$

where *T* is the translational vector *T* mapping C, the optical center, to a point in the world reference frame $F_{W}$, and *R* is the rotation matrix, using the [zyx] convention [35]:

(5) $$R=\left[\begin{array}{ccc} c_{\psi }c_{\theta } & c_{\psi }s_{\phi }s_{\theta }-c_{\phi }s_{\psi } & s_{\phi }s_{\psi }+c_{\phi }c_{\psi }s_{\theta }\\ c_{\theta }s_{\psi } & c_{\phi }c_{\psi }+s_{\phi }s_{\psi }s_{\theta } & c_{\phi }s_{\psi }s_{\theta }-c_{\psi }s_{\phi }\\ -s_{\theta } & c_{\theta }s_{\phi } & c_{\phi }c_{\theta }, \end{array}\right],$$

where s=sin, c=cos, and ${\left[\phi ,\theta ,\psi \right]}^{T}$ represent the attitude angles, or Euler angles.

#### 5.2.2. Intrinsic Parameters

From the pinhole camera model used in this work, there are three intrinsic parameters that need to be determined:

1. The focal length *f*.
2. Transformation between the camera frame coordinates and the pixel coordinates.
3. Geometric distortion caused by the camera optics.

The focal length is defined in pixels, and in some cases, it is supplied by the manufacturer. In our case, the parameters were not supplied by the manufacturer, and f in pixels was identified in [36] and is shown in Table 1.

Since the image plane is measured in pixels we need to introduce scaling factors $k_{u},k_{v}$, which have units in $\frac{pixel}{mm}$ to perform a transformation between the camera frame coordinates and the pixel coordinates. In addition, $k_{u},k_{v}$ are not identical due to imperfections in the camera [39]. Given that the pixel size is unknown and that we do not have intrinsic values for $k_{u},k_{v}$, these values were obtained by experimentation. The values are shown in Table 1. Now by rescaling the u,v axes in the image plane we get:

(6) $$\begin{array}{c} u_{p}=k_{u}\frac{f}{Z}X_{P}u_{0},\\ v_{p}=k_{v}\frac{f}{Z}Y_{P}v_{0}, \end{array}$$

where ${\left[u_{0},v_{0}\right]}^{T}$ are the principal point coordinates.

In the current work we assumed, (i.e., assumption 2), that the camera has no distortion and thus we ignore its effect on the image and its intrinsic parameters.

### 5.3. Stereo Vision

**Figure 4:**
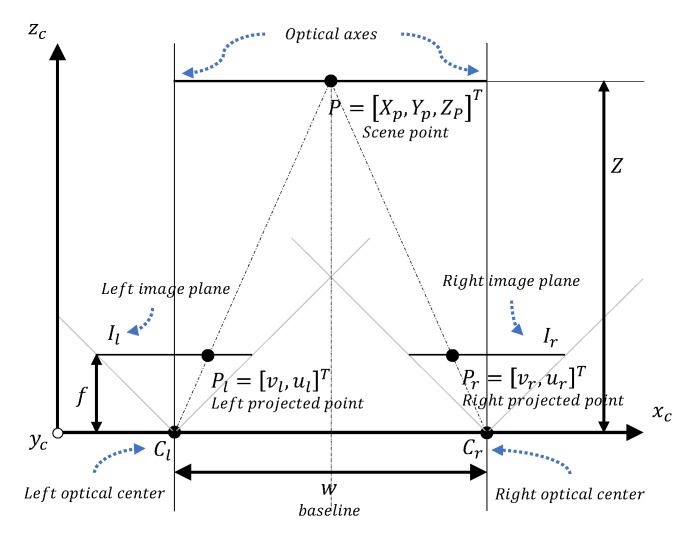
Stereo triangulation.

After performing camera calibration, the second objective is to compute the distance *Z* from the drone to the object, which coincides with the line going from the middle of the two optical centers ${\left[C_{l},C_{r}\right]}^{T}$, to the scene point *P*, as shown in Figure 4.

The chosen method for finding *Z* is stereo vision or stereopsis, in which depth information is derived from two separate views. In our case from two cameras with optical centers ${\left[C_{l},C_{r}\right]}^{T}$, where subscript ${}_{l}{,}_{r}$ indicates the left and right camera respectively. This principle involves calibrating the cameras, establishing the correspondences from both image planes, and then computing the 3D coordinates of the scene points [33].

### 5.4. Coordinate Computation with Rectified Configuration

Our system setup has a rectified camera configuration, also referred to as canonical configuration, where image planes (epipolar lines) coincide and are parallel with the lines going trough ${\left[C_{l},C_{r}\right]}^{T}$ in the $x_{c}$ axis in Figure 4. Thus the two optical lines are parallel. This method differs from the general stereo camera configuration where the epipolar planes do not coincide. According to this, the orthogonal distance from the $x_{c}$ axis to the *P* is *Z*. In Figure 4, the distance between the two optical centers ${\left[C_{l},C_{r}\right]}^{T}$*w*, is referred as the baseline. If assumption 5 holds, then both cameras will have a line of sight to the scene point $P={\left[X,Y,Z\right]}^{T}$, which will be projected onto the two image planes ${\left[I_{l},I_{r}\right]}^{T}$, where the projection is referred to as ${\left[P_{l},P_{r}\right]}^{T}$ and $v_{r}=v_{l}$.

**Figure 5:**
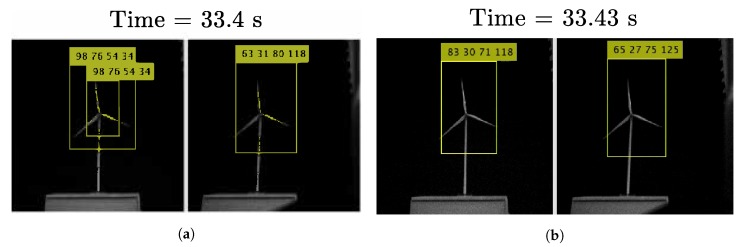
Experiment with the drone stationary positioned 750 mm in front of the wind turbine. (**a**) Image from left and right camera, frame 1; (**b**) Image from left and right camera, frame 2.

In Figure 5a, we can see the projection of the wind turbine in the left and the right image. In that figure, the center of the bounding box ${\left[b_{x},b_{y}\right]}^{T}$ is equivalent to ${\left[P_{l},P_{r}\right]}^{T}$.

From here we can calculate the projection disparity, which is the difference in the distance of the two projections:

(7) $$D=v_{r}-v_{l}$$

Using the geometry of right-angled triangles the distance *Z* can be found through the following equation:

(8) $$\frac{w}{Z}=\frac{D}{f}\Rightarrow Z=\frac{fw}{D}.$$

From here we can see that the distance *Z* is inversely proportional to the disparity, and proportional to *w*. We can also infer that:

(9) (ifD→0thenZ→∞),

and as D→0 the error $e=Z-Z_{true}$ increases.

### 5.5. Correspondence Problem

The challenge of the correspondence problem is to identify the same two points in an object from two images, taken of the same scene [39]. Our solution to the correspondence problem is to use YOLO to compute the disparity, using the following equation

(10) $$\begin{array}{c} v_{l}=l\left(b_{x}\right)\\ v_{r}=r\left(b_{x}\right), \end{array}$$

where $l(b_{x})$ and $r(b_{x})$ are the center *x* coordinate $b_{x}$ of the left and right images bounding box respectively. The calculation of $b_{x}$ is shown in Equation (2).

### 5.6. Signal Analysis and Processing

**Figure 6:**
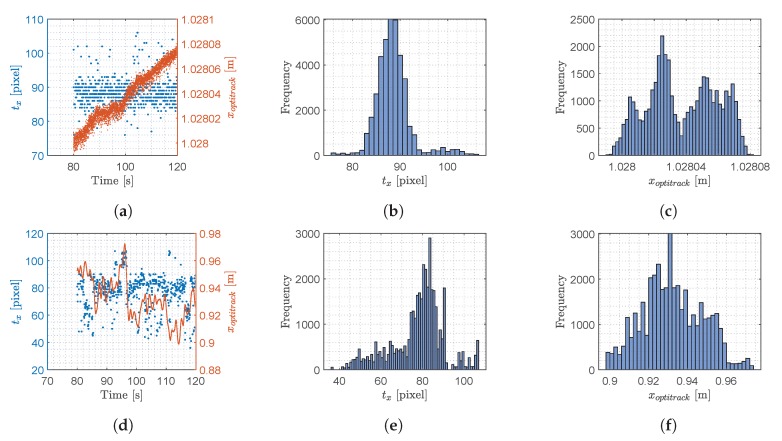
Results from the two experiments where the drone was stationary and in steady flight, this data is with respect to *t_x_*. (**a**) Stationary test, raw data of *t_x_*; (**b**) Stationary test, histogram of *t_x_*; (**c**) Stationary test, histogram of *x_optitrack_*; (**d**) Steady flight test, raw data of *t_x_*; (**e**) Steady flight test, histogram of *t_x_*; (**f**) Steady flight test, histogram of *x_optitrack_*.

**Figure 7:**
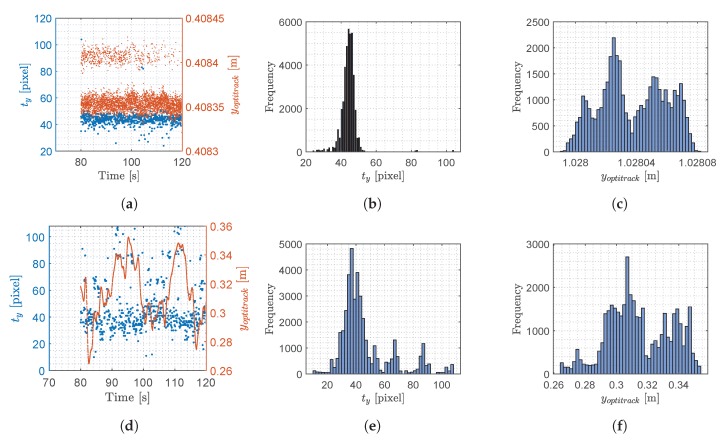
Results from the two experiments where the drone was stationary and in steady flight, this data is with respect to *t_y_*. (**a**) Stationary test, raw data of *t_y_*; (**b**) Stationary test, histogram of *t_y_*; (**c**) Stationary test, histogram of *y_optitrack_*; (**d**) Steady flight test, raw data of *t_y_*; (**e**) Steady flight test, histogram of *t_y_*; (**f**) Steady flight test, histogram of *y_optitrack_*.

In this section the signal generated by YOLO and used for computing the object position in the 3D space will be analyzed to investigate its statistical features and frequency domain properties. The output signal from each of the two YOLO networks attached to the stereo camera consists of four variables, ${\left[t_{x}t_{y}t_{w}t_{h}\right]}^{T}$. In this work, we chose to show the analysis of $t_{x}$ and $t_{y}$ only, as the other variables exhibit similar features. Since the output signal generated by YOLO changes and does not have stationary statistical features, the analysis will be based on signals obtained at different operating conditions. Two experiments were considered: (i) stationary test, (ii) steady flight. The results are plotted in Figure 6 and Figure 7 for $t_{x}$ and $t_{y}$ respectively, the optitrack position is plotted as well, to illustrate the precise movements of the drone during the experiments.

From the Figure 6 and Figure 7, a clear pattern emerges. When the drone is stationary, the distribution is narrower albeit with outliers in comparison to the flying scenario, where the histogram shows several peaks. The data distribution of the stationary experiment has some resemblance to a normal distribution except for some outliers. These outliers are caused when YOLO detects different sizes of bounding boxes for the same image, a phenomenon which is illustrated in Figure 5. In this figure, the image from the left camera shows a double bounding box as illustrated in Figure 5a. Fortunately, the effect of this error produced occasionally by YOLO, is minimum because the center of the bounding box as calculated in Equation (2) shows little variation when two bounding boxes are generated by YOLO.

#### 5.6.1. Frequency Spectrum and Filtering

**Figure 8:**
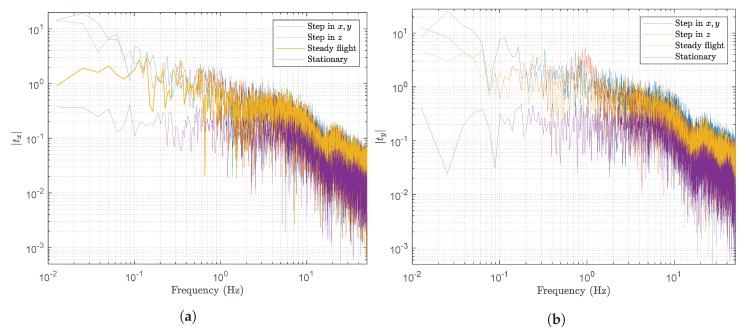
Signal analysis. (**a**) FFT of four different experiments wrt. *t_x_*; (**b**) FFT of four different experiments wrt. *t_y_*.

The signals generated by YOLO are not the kind of signals produced by typical sensors, because they are generated by the processing applied in the multiple layers of YOLO. Additionally, they showed non-normal statistical features. Hence, prior to use them to computing the ${\left[X,Y,Z\right]}^{T}$ coordinates of a real object, they must be filtered. In this section, we analyze the YOLO’s signals using the fast Fourier transform (FFT). The signal’s frequency characteristics are shown in Figure 8, where Figure 8a,b are the the FFTs of $t_{x}$ and $t_{y}$ respectively, from four different experiments: (i) step in ${\left[x,y\right]}^{T}$, (ii) step in *z*, (iii) steady flight and (iv) stationary.

From these figures, it is clear that low frequencies are introduced when the drone is moving. However, when it is stationary, the signals produced have no low frequency components. However, since these signals will be used as feedback to control drone’s movement in a future work, we want to keep their main dynamic frequency features, which are located approximately below 0.1 Hz.

#### 5.6.2. Filter Design

Two filters were designed to process YOLO’s signals, one for the *X* and *Y* computation and one for the *Z* computation. The reason is that the computation of *Z*, involves the disparity parameter *D*, which depends on a ratio of $b_{x}$ from both the left and right cameras, thus is more prone to the noise generated by YOLO. Therefore, the same filter used for the processing of ${\left[X,Y\right]}^{T}$ did not perform well in the case of *Z*. For ${\left[X,Y\right]}^{T}$ a low-pass infinite impulse response (IIR) filter with a cut frequency of 0.5 Hz was designed, and a low-pass IIR filter with a cut-off frequency of 0.03 Hz was used for processing *Z*. The design of the filter for *Z* signal was fine-tuned, to improve sensor’s performance.

## 6. Implementation

**Figure 9:**
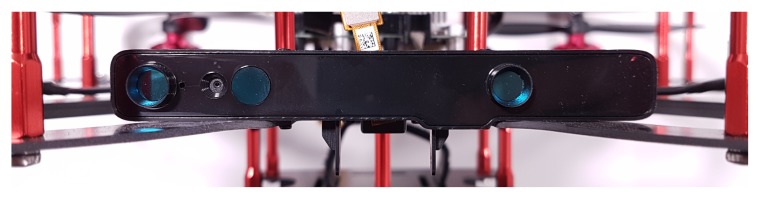
Intel RealSense R200 Camera mounted onto the Quanser Q-Drone.

Our implementation and testing platform was a Quanser’s AVRS system [40], which contains a quad-copter, the Qdrone, a ground control station and a six-camera optitrack flex13 array used for motion capture [41]. The cameras used in this work are intel’s RealSense Stereoscopic Depth Camera (R200) [38], shown in Figure 9. This camera system has two identical left-right 10bit IR cameras placed 70 mm apart with a global shutter and a video graphics array (VGA) resolution (640 × 480). The cameras are complementary metal–oxide–semiconductor (CMOS) monochrome with a cut filter blocking out wavelengths below 840 nm. The camera has a field of vision (FOV) of, 60${}^{{}^{\circ}}$ × 45${}^{{}^{\circ}}$ × 70${}^{{}^{\circ}}$, in the horizontal, vertical and diagonal direction respectively [37]. The drone is also equipped with a monocular red green blue (RGB) camera and a laser projector, but these units were not used in this work.

As our DNN is trained for a RGB image, in the current version we convert the gray-scale image into a RGB image by replicating the gray-scale for each color channel and feed it into the DNN.

## 7. Experiments and Results

**Figure 10:**
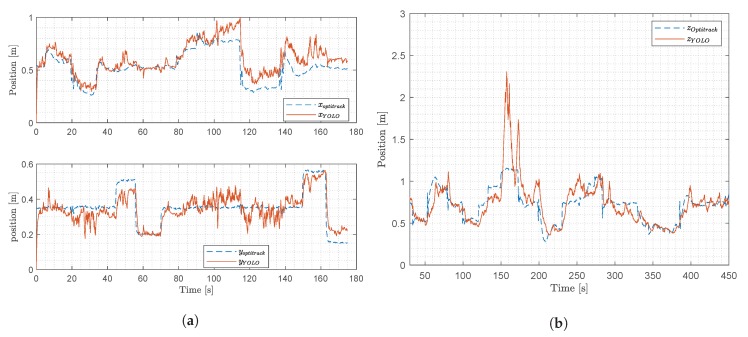
Results from experiments one and two, showing the computed positions and the measured optitrack positions. (**a**) Results from experiment one; (**b**) Results from experiment two.

In this paper we propose a method to estimate the ${\left[X,Y,Z\right]}^{T}$ coordinates of an object in a 2D scene, using a stereo camera with two YOLO DNNs attached. These DNNs were trained to detect the position of a wind turbine’s blades within an image. The method we have designed was evaluated in a laboratory setup, using an experimental platform described in Section 6. During both experiments, the drones are sat to track the desired reference, which is carefully designed to test the system. Two experiments were performed as described below:

**Experiment** **1:** to evaluate drone’s position calculation using the perspective projection, the drone is moved in the X,Y direction with a range of distances. Our aim is to explore the entire FOV of the camera in this test, i.e., move the drone such that the object is projected in all corners of the FOV. The results are shown in Figure 10a are from the same experiment at the same time frame.
**Experiment** **2:** the drone is moved in the *Z* direction to evaluate the sensor’s depth estimation calculation using stereo triangulation. In this experiment, we moved the drone within a range of displacements, aiming at finding the boundaries of the method. This was done moving the drone very close and far away from the target object.

The computed positions of the two experiments together with the optitrack position data are shown in Figure 10a,b for the experiment one and two respectively.

### 7.1. Result Discussion

#### 7.1.1. Results of the *X* and *Y* Position

**Figure 11:**
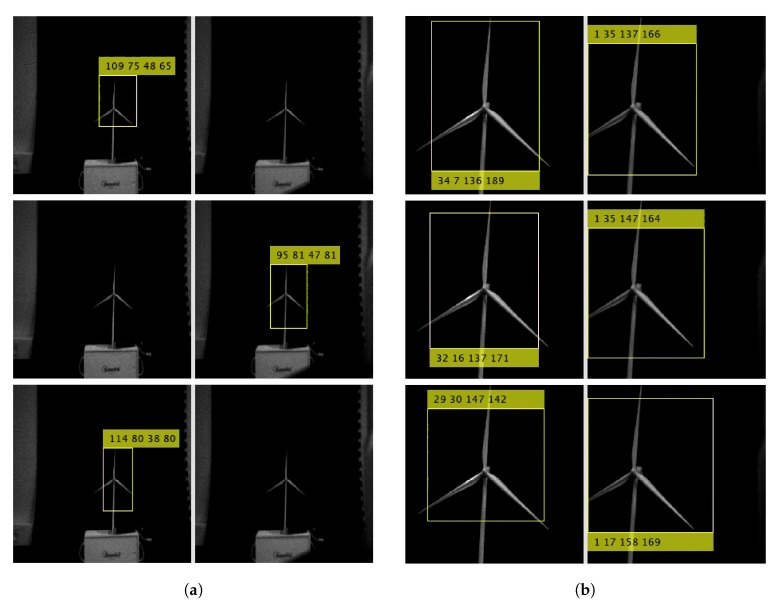
Images captured by the drone’s stereo IR camera. (**a**) Three frames from left and right camera, when the drone is furthest away form the wind turbine, equivalent to 150 s in Figure 10b; (**b**) Three frames from left and right camera, when the drone is nearest to the wind turbine, equivalent to 210 s in Figure 10b.

During the experiments, we observed that the computation of *Z* works but only under certain conditions, i.e., when assumption 5 is satisfied. When, in the course of experiment one, the drone was moved in the *X* and *Y* direction, in certain occasions the view of the wind turbine was lost from one of the cameras. The phenomenon is illustrated in Figure 11a. This issue induced errors in the computation of *Z*, as Equation (8) produced a division by 0. Thus, for the computation of X,Y (in Equation (6)), and shown in Figure 10, the distance obtained from the optitrack signal was used.

YOLO’s computed *X* position follows the optitrack signal measured accurately, as is shown on the top plot in Figure 10a. The YOLO’s computed *Y* position shown on the bottom plot of Figure 10a, indicates that this signal too has relatively good tracking of the optitrack signal. This figure also shows that in the steady flight experiment, at a time interval t=[70−150] s, the computation of the signal $y_{YOLO}$ presents some variations. This is due to the drone’s movement in the *X* direction that can be seen on the top subplot in the same figure. The experiment indicates that the drone’s movement in one direction has some effect on the computed position in the other direction. Furthermore, if the drone’s movement pushes the bounding box towards the edge of the image, the measured variations become even larger.

#### 7.1.2. Results for the *Z* Position

The distance calculation from the drone to the wind turbine performed well in general. However, our experiments also show that there is a large deviation at 150 s in Figure 10b. This occurs when the drone is farthest away from the wind turbine and the DNNs cannot detect it anymore. This phenomenon was also observed in other experiments when the wind turbine was far away from the drone. This is a known issue of YOLO, that as has been reported in [42], has problems detecting small objects.

This effect is shown in Figure 11a containing three frames captured with the left and right camera and that were taken when the drone was farthest away from the wind turbine blades. The left and right images alternate in failing to recognize the wind turbine blades. This is caused by the large error in position’s computation at 150 s in Figure 10b.

As a comparison, Figure 11b illustrates three frames captured by the left and right camera when the drone is closest to the wind turbine, three frames are captured at 210 s according to Figure 10b.

## 8. Discussion

The sensor described in this paper may be used to determine the position of any object in a 3D space. However, the DNNs should be retrained to being able to detect the new object. In this work, the object chosen was a wind turbine, because our ultimate goal is to use this sensor in the vision-based servoing system in a drone, specially designed to perform wind turbine inspections autonomously.

The method used for computing drone’s relative position to the wind turbine’s blades worked successfully within the confined space of our laboratory. The sensor achieved good tracking performance in all three axis, as is described in Section 7.

The measurements used to evaluate sensor performance, were obtained when the drone was flying. We observed that even if the drone was not perfectly stable all the time, but moving slightly, our sensor still produced accurate results.

We noticed that when YOLO detects a wind turbine, the generated signals for [X,Y,Height,Width] positions and sizes of the bounding boxes are noisy. Additionally YOLO produces on occasions double bounding boxes. These issues had to be handled by using appropriate filtering techniques.

We also observed that an important issue in this type of DNN-based sensor is the resolution of the images used for training. This factor has a direct effect on object detection accuracy. In this work, the DNNs were trained with images of a relatively low resolution (224×224) to reduce training and inference time. However, as is discussed in Section 7.1, this had the effect that when the drone was at a relatively long distance from the wind turbine, the DNNs were not able to detect it. One way to solve this problem is to increase image resolution and add digital zooming, but this will require more memory and the use of high-performance GPUs or TPUs in the implementation of the sensor on a drone.

## 9. Conclusions

We have designed a visual sensor based on a stereo camera and two DNNs that is capable of measuring accurately the position of a wind turbine in a 3D space.

To implement the sensor some challenges had to be overcome. First, we observed that the signals computed by YOLO to determine the size and location of the bounding boxes on wind turbines had a significant spread in its random distribution. This is due to the object detection algorithm in YOLO that is constantly analyzing an image to detect and determine the location of the wind turbine, generating in this process some randomness in the data produced.

Secondly, these noisy signals had to be filtered before they could be used for the computation of the *X* and *Y* coordinates of the target object. Furthermore, appropriate filtering of these signals was essential, given that the computation of *Z* depends on the left and right object detection signals produced by the two YOLO DNNs.

One of the issues that motivated this work, was the problem of finding new ways to achieving a higher level of autonomy on a UAV designed for inspection tasks, using only vision sensors. This work and the current state of the art literature reviewed in Section 2, shows that vision can be used effectively for some of the key tasks in autonomous robotics, such as visual servoing, drone’s localization, damage detection, path planning and autonomous take-off and landing. However, some of these techniques still use other types of sensors combined with vision.

An open research question that was left for future research work is to determine what is the optimal image resolution that should be used for training and inferencing in a DNN especially designed for wind turbine detection. Larger resolution images will require larger networks to enable the DNNs achieve better detection accuracy at long distances, at the cost of increasing inference time. Smaller images reduce training time and enable the DNNs to learn the main global features of an object, allowing them to perform inferences faster. This is done at the expense of discarding finer features that may enable a DNN to distinguish a particular object from other similar objects. Our goal is to use the sensor signals in the feedback loop of a visual servoing system to control drone’s navigation towards the wind turbine’s hub. Therefore, we will have limitations in terms of memory and processing power if larger networks are used. DNNs such as TinyYolo may help in this aspect at the cost of getting reduced detection accuracy.

Finally, to avoid the manual labeling of images with bounding boxes, we will also look at the problem of labeling objects automatically using computer-aided simulation of 3D objects. This system will enable us to keep programmatic control of an object and of the initial bounding box that will be inserted manually in the beginning. The movements of the virtual camera could be tracked to compute automatically the position and sizes of the new bounding boxes. This information will be recorded and used to create a labeled training set.

## Figures and Tables

**Table 1:** System Parameters.

Parameter	Value	Unit	Reference
*f* “right camera”	581.48,586.05	pixels	[36]
*f* “left camera”	575.79,579.88	pixels	[36]
$k_{u}$	0.015	pixels/mm	
$k_{v}$	0.009	pixels/mm	
w	70	mm	[37]
Camera resolution	640 × 480	pixels	[38]

## References

[1] Vaillant R., Monrocq C., LeCun Y. (1994). Original approach for the localisation of objects in images. IEE Proc.-Vision Image Signal Process..

[2] Viola P., Jones M. Rapid object detection using a boosted cascade of simple features. Proceedings of the 2001 IEEE Computer Society Conference on Computer Vision and Pattern Recognition, CVPR 2001.

[3] Dalal N., Triggs B. Histograms of oriented gradients for human detection. Proceedings of the 2005 IEEE Computer Society Conference on Computer Vision and Pattern Recognition (CVPR’05).

[4] Girshick R.B., Donahue J., Darrell T., Malik J. (2013). Rich feature hierarchies for accurate object detection and semantic segmentation. arXiv.

[5] Lin T., Goyal P., Girshick R.B., He K., Dollár P. (2017). Focal Loss for Dense Object Detection. arXiv.

[6] Redmon J., Divvala S.K., Girshick R.B., Farhadi A. (2015). You Only Look Once: Unified, Real-Time Object Detection. arXiv.

[7] Redmon J., Farhadi A. (2016). YOLO9000: Better, Faster, Stronger. arXiv.

[8] Redmon J., Farhadi A. (2018). YOLOv3: An Incremental Improvement. arXiv.

[9] Liu W., Anguelov D., Erhan D., Szegedy C., Reed S.E., Fu C., Berg A.C. (2015). SSD: Single Shot MultiBox Detector. arXiv.

[10] Huang B., Zhao J., Liu J. (2019). A Survey of Simultaneous Localization and Mapping. arXiv.

[11] Thrun S., Burgard W., Fox D. (2005). Probabilistic Robotics.

[12] Chaumette F., Hutchinson S. (2006). Visual servo control. I. Basic approaches. IEEE Robot. Autom. Mag..

[13] Giusti A., Guzzi J., Ciresan D.C., He F.L., Rodríguez J.P., Fontana F., Faessler M., Forster C., Schmidhuber J., Di Caro G. (2016). A Machine Learning Approach to Visual Perception of Forest Trails for Mobile Robots. IEEE Robot. Autom. Lett..

[14] Padhy R.P., Verma S., Ahmad S., Choudhury S.K., Sa P.K. (2018). Deep Neural Network for Autonomous UAV Navigation in Indoor Corridor Environments. Procedia Comput. Sci..

[15] Loquercio A., Maqueda A.I., del Blanco C.R., Scaramuzza D. (2018). Dronet: Learning to fly by driving. IEEE Robot. Autom. Lett..

[16] Smolyanskiy N., Kamenev A., Smith J., Birchfield S. (2017). Toward low-flying autonomous MAV trail navigation using deep neural networks for environmental awareness. arXiv.

[17] Bojarski M., Del Testa D., Dworakowski D., Firner B., Flepp B., Goyal P., Jackel L.D., Monfort M., Muller U., Zhang J. (2016). End to end learning for self-driving cars. arXiv.

[18] Muller U., Ben J., Cosatto E., Flepp B., Cun Y.L. Off-road obstacle avoidance through end-to-end learning. Proceedings of the 18th International Conference on Neural Information Processing Systems.

[19] Chen Z., Huang X. End-to-end learning for lane keeping of self-driving cars. Proceedings of the 2017 IEEE Intelligent Vehicles Symposium (IV).

[20] Bojarski M., Yeres P., Choromanska A., Choromanski K., Firner B., Jackel L., Muller U. (2017). Explaining how a deep neural network trained with end-to-end learning steers a car. arXiv.

[21] Sans-Muntadas A., Kelasidi E., Pettersen K.Y., Brekke E. (2019). Learning an AUV docking maneuver with a convolutional neural network. IFAC J. Syst. Control..

[22] Drews P., Williams G., Goldfain B., Theodorou E.A., Rehg J.M. (2019). Vision-Based High-Speed Driving with a Deep Dynamic Observer. IEEE Robot. Autom. Lett..

[23] Durdevic P., Ortiz-Arroyo D., Li S., Yang Z. (2019). Vision Aided Navigation of a Quad-Rotor for Autonomous Wind-Farm Inspection.

[24] Goldfain B., Drews P., You C., Barulic M., Velev O., Tsiotras P., Rehg J.M. (2019). AutoRally: An Open Platform for Aggressive Autonomous Driving. IEEE Control. Syst. Mag..

[25] Gemerek J., Ferrari S., Wang B.H., Campbell M.E. (2019). Video-guided Camera Control for Target Tracking and Following. IFAC-PapersOnLine.

[26] Howard A.G., Zhu M., Chen B., Kalenichenko D., Wang W., Weyand T., Andreetto M., Adam H. (2017). MobileNets: Efficient Convolutional Neural Networks for Mobile Vision Applications. arXiv.

[27] Li P., Chen X., Shen S. (2019). Stereo R-CNN based 3D Object Detection for Autonomous Driving. arXiv.

[28] Bateux Q., Marchand É., Leitner J., Chaumette F. (2017). Visual Servoing from Deep Neural Networks. arXiv.

[29] Krizhevsky A., Sutskever I., Hinton G.E. Imagenet classification with deep convolutional neural networks. Proceedings of the Advances in Neural Information Processing Systems.

[30] Shao L., Zhu F., Li X. (2015). Transfer Learning for Visual Categorization: A Survey. IEEE Trans. Neural Netw. Learn. Syst..

[31] Durdevic P., Ortiz-Arroyo D., Li S., Yang Z. UAV Visual Servoing Navigation in Sparsely Populated Environments. Proceedings of the 15th European Workshop on Advanced Control and Diagnosis, ACD 2019.

[32] Holzmann C., Hochgatterer M. Measuring Distance with Mobile Phones Using Single-Camera Stereo Vision. Proceedings of the 2012 32nd International Conference on Distributed Computing Systems Workshops.

[33] Sonka M., Hlavac V., Boyle R. (2014). Image Processing, Analysis, and Machine Vision.

[34] Trucco E., Verri A. (1998). Introductory Techniques for 3-D Computer Vision.

[35] Fossen T.I. (1994). Guidance and Control of Ocean Vehicles.

[36] Yang K., Wang K., Zhao X., Cheng R., Bai J., Yang Y., Liu D. (2017). IR stereo RealSense: Decreasing minimum range of navigational assistance for visually impaired individuals. J. Ambient. Intell. Smart Environ..

[37] Keselman L., Iselin Woodfill J., Grunnet-Jepsen A., Bhowmik A. Intel realsense stereoscopic depth cameras. Proceedings of the IEEE Conference on Computer Vision and Pattern Recognition Workshops.

[38] Intel (2019). Intel RealSense Camera R200. https://ark.intel.com/content/www/us/en/ark/products/92256/intel-realsense-camera-r200.html.

[39] Siegwart R., Nourbakhsh I.R., Scaramuzza D. (2011). Introduction to Autonomous Mobile Robots.

[40] Quanser (2019). Autonomous Vehicles Research Studio. https://www.quanser.com/products/autonomous-vehicles-research-studio/.

[41] OptiTrack (2019). Flex 13. https://optitrack.com/products/flex-13/.

[42] Liu L., Ouyang W., Wang X., Fieguth P., Chen J., Liu X., Pietikäinen M. (2020). Deep Learning for Generic Object Detection: A Survey. Int. J. Comput. Vis..

